# Deletion of both Dectin-1 and Dectin-2 affects the bacterial but not fungal gut microbiota and susceptibility to colitis in mice

**DOI:** 10.1186/s40168-022-01273-4

**Published:** 2022-06-14

**Authors:** Yazhou Wang, Madeleine Spatz, Gregory Da Costa, Chloé Michaudel, Alexia Lapiere, Camille Danne, Allison Agus, Marie-Laure Michel, Mihai G. Netea, Philippe Langella, Harry Sokol, Mathias L. Richard

**Affiliations:** 1 Universite Paris-Saclay, INRAE, AgroParisTech, Micalis Institute, Domaine de Vilvert, 78352 Jouy en Josas, France; 2 Paris Center for Microbiome Medicine, Fédération Hospitalo-Universitaire, 75012 Paris, France; 3 Department of Internal Medicine and Center for Infectious Diseases, Radboud University, Nijmegen, Netherlands; 4 Department of Immunology and Metabolism, Life & Medical Sciences Institute, University of Bonn, Bonn, Germany; 5 Gastroenterology Department INSERM, Centre de Recherche Saint-Antoine, CRSA, AP-HP, Saint Antoine Hospital, Sorbonne Université, F-75012 Paris, France

**Keywords:** Microbiota, Gut inflammation, Dectin-1, Dectin-2, Immune response

## Abstract

**Background:**

Innate immunity genes have been reported to affect susceptibility to inflammatory bowel diseases (IBDs) and colitis in mice. Dectin-1, a receptor for fungal cell wall β-glucans, has been clearly implicated in gut microbiota modulation and modification of the susceptibility to gut inflammation. Here, we explored the role of Dectin-1 and Dectin-2 (another receptor for fungal cell wall molecules) deficiency in intestinal inflammation.

**Design:**

Susceptibility to dextran sodium sulfate (DSS)-induced colitis was assessed in wild-type, Dectin-1 knockout (KO), Dectin-2KO, and double Dectin-1KO and Dectin-2KO (D-1/2KO) mice. Inflammation severity, as well as bacterial and fungal microbiota compositions, was monitored.

**Results:**

While deletion of Dectin-1 or Dectin-2 did not have a strong effect on DSS-induced colitis, double deletion of Dectin-1 and Dectin-2 significantly protected the mice from colitis. The protection was largely mediated by the gut microbiota, as demonstrated by fecal transfer experiments. Treatment of D-1/2KO mice with opportunistic fungal pathogens or antifungal agents did not affect the protection against gut inflammation, suggesting that the fungal microbiota had no role in the protective phenotype. Amplicon-based microbiota analysis of the fecal bacterial and fungal microbiota of D-1/2KO mice confirmed the absence of changes in the mycobiota but strong modification of the bacterial microbiota. We showed that bacteria from the *Lachnospiraceae* family were at least partly involved in this protection and that treatment with *Blautia hansenii* was enough to recapitulate the protection.

**Conclusions:**

Deletion of both the Dectin-1 and Dectin-2 receptors triggered a global shift in the microbial gut environment, affecting, surprisingly, mainly the bacterial population and driving protective effects in colitis. Members of the *Lachnospiraceae* family seem to play a central role in this protection. These findings provide new insights into the role of the Dectin receptors, which have been described to date as affecting only the fungal population, in intestinal physiopathology and in IBD.

Video Abstract

**Supplementary Information:**

The online version contains supplementary material available at 10.1186/s40168-022-01273-4.

## Background

Scientific findings over the last decade have demonstrated that the impact of the gut microbiota on human health is considerable but that many functions remain to be uncovered. A tremendous amount of data on the bacterial portion of the microbiota is accumulating since this microbial population is by far the largest one in the gut. Nevertheless, the scientific community is now widening the scope of their investigations, and novel data on other components of the human microbiota (bacteriophages, viruses, archaea, fungi, etc.) are appearing in the literature. Fungi, although present in relatively low numbers, are significant components of the ecosystem and thus part of its equilibrium, and research on fungi has therefore gradually emerged as a new area in microbiota investigation. Fungal cells are 10-fold longer and 100-fold larger in volume than bacterial cells, suggesting that fungal biomass and fungal-derived metabolites cannot be compared to bacteria by considering cell counts alone. To date, many results have pointed to a potential link between the fungal gut microbiota and inflammatory bowel diseases (IBDs), such as Crohn’s disease and ulcerative colitis. For instance, by monitoring the bacterial and fungal compositions of (i) the fecal microbiota of patients with IBD and (ii) the mucosa-associated microbiota of patients with Crohn’s disease, we recently showed the occurrence of fungal dysbiosis similar to bacterial dysbiosis during IBD flare-ups, with alterations in the biodiversity and composition of the mycobiota [1, 2]. An increasing amount of data has recently been produced regarding the crosstalk between the host and the mycobiota through the different actors of the host immune system. First, among the genetic polymorphisms associated with IBD, single-nucleotide polymorphisms (SNPs) were detected in CARD9, a cytoplasmic protein that plays a central role in integrating signals downstream of fungus-recognizing receptors such as Dectin-1 and Dectin-2. Our group previously showed that mice devoid of CARD9 were more sensitive to colitis and had an increased intestinal fungal burden, but antifungal treatment reduced this hypersensitivity [3, 4]. We also demonstrated in a recent publication that bacterial-fungal interactions are central in the development of gut inflammation, particularly with regard to the effect of fungi on this inflammation [5]. Since then, further research has been conducted on the involvement of Dectin-1 and Dectin-2 receptors in the crosstalk with the host, especially regarding their impact on susceptibility to gut inflammation. Dectin-1 and Dectin-2 are two C-type-like receptors described for their capacity to recognize the fungal cell wall ligands beta-glucan and mannans, respectively. The literature on Dectin-1 is controversial since different publications showed opposite behaviors of Dectin-1KO mice in a gut inflammation model. While Iliev et al. presented increased susceptibility of Dectin-1KO mice, Tang and coworkers observed protection [3]. Interestingly, further analysis of both works by Iliev in a following comment letter led to the proposal of a model to reconcile the two studies. In this comment, the author suggested that the basal composition of the fungal microbiota was crucial to the outcome of colitis [6]. In the presence of opportunistic fungi, the absence of Dectin-1 results in fungal overgrowth, leading to the participation of these fungi in increasing gut inflammation [6]. In Tang et al.’s work, in the absence of opportunistic fungi, a completely different mechanism was proposed, involving decreased levels of the antimicrobial peptides (AMPs) S100a8 and S100a9, impacting the bacterial microbiota and particularly the overgrowth of *Lactobacillus murinus* in the absence of Dectin-1, which has a positive effect on Treg cells and consequently on colitis development [7]. Another publication by Heinsbroek et al. showed no effect of Dectin-1 deletion on mouse susceptibility in two models of gut inflammation and infection with *Helicobacter hepaticus* [8]. These publications suggest that the effect of Dectin-1 is greatly impacted by other parameters, one of them being the basal mycobiota and the others being unidentified thus far. Nothing is known about the effect of Dectin-2 in colitis, although several studies have been published on its role in fungal infections [9, 10].

Here, we studied the effect of Dectin-2 deletion as well as that of double deletion of Dectin-1 and Dectin-2 on gut inflammation in a DSS-induced colitis model. We showed that the Dectin-1 and Dectin-2 double knockout (D-1/2KO, produced for this work) was strongly protected from gut inflammation and that this protection was mainly driven by modification of the bacterial gut microbiota and not the mycobiota. Further analysis allowed the identification of potential probiotic bacteria, mainly from the *Lachnospiraceae* family, such as *Blautia hansenii*, which showed a protective effect when administered to mice before and during DSS-induced colitis.

## Material and methods

### Mice

Dectin-1-deficient mice (D-1KO) were kindly provided by Gordon D. Brown, and Dectin-2-deficient mice (D-2KO) were kindly provided by Mihai G. Netea; these mice were used to establish our colonies of dectin-KO mice, including Dectin-1 and Dectin-2 double-deficient mice (Dectin-1/2 deficient, D-1/2KO) and control littermates animals (wild-type—WT). These mice were generated in a C57BL/6 background and included in the protocol at 8 weeks old. Eight-week-old female wild-type mice were purchased from Janvier Laboratory (Le Genest, France) and used 1 week after reception (WTj). Animals were kept in humidity- and temperature-controlled rooms under a 12-h light-dark cycle and had access to a chow diet and water ad libitum. All experiments were performed with appropriate control groups from the same batch of mice. All experiments were performed in accordance with the ethics committee “Comite d’Ethique en Experimentation Animale” (COMETHEA C2EA – 45, Jouy en Josas, France). Every experiment was repeated at least two times *n* = 10.

### Genotyping of Dectin-1 and Dectin-2 mutants

Mouse ear samples were collected. Ear DNA was extracted according to the recommended method. Briefly, 300 μL of lysis buffer (50 mM Tris-HCl 1 M pH 7.8 (Sigma–Aldrich, St. Louis, MO, USA), 100 mM NaCl (Sigma–Aldrich, St. Louis, MO, USA), 0.5% Tween 20 (Sigma–Aldrich, St. Louis, MO, USA)), and 1.5 μL of proteinase K (Sigma–Aldrich, St. Louis, MO, USA) were added to the tube containing mouse tissue and mixed well. The mixture was incubated overnight at 56 °C with agitation until a homogeneous solution was obtained. The solution was next incubated at 95 °C for 20 min to inactivate proteinase K and then centrifuged at 10,000 × *g* for 5 min. Genomic DNA was recovered from the supernatant and stored at − 20 °C. Subsequently, the DNA was amplified using Dectin-1 and Dectin-2 primers, as shown in Supplemental Table 1. The PCR products were then run on a 1.5% agarose gel to confirm the mouse genotype.

### Induction of colitis with Dextran Sodium Sulfate (DSS)

Mice were given 2% (wt/vol) DSS (molecular weight, 36,000–50,000; MP Biomedicals, Solon, OH) dissolved in drinking water ad libitum for 7 days, followed by a recovery period (water only) of 5 days. Animals were monitored daily for weight loss and disease activity index (including three parameters: weight loss, stool consistency, and presence of blood in feces).

### Fecal Microbiota Transfer (FMT)

Microbiota transfer was performed by fecal gavage using a modified version of a previously described protocol [11] as follows: fresh stool samples were recovered from 10 mice (WTj or D-1/2KO mice) and immediately stored in an anaerobiosis generator (Genbox, Biomérieux, Capronne, France) to favor the preservation of anaerobic bacteria. The samples were processed within 6 h in an anaerobic chamber. The feces were rapidly diluted 100-fold in LYHBHI (brain–heart infusion) medium (BD Difco, Le Pont De Claix, France) supplemented with cellobiose (1 mg/ml; Sigma–Aldrich, St. Louis, MO, USA), maltose (1 mg/ml; Sigma–Aldrich), and cysteine (0.5 mg/ml; Sigma–Aldrich). This ready-to-use fecal suspension was used for FMT to mice.

Mice were fasted for 1 h and then subjected to bowel cleansing by oral-gastric gavage with PEG (polyethylene glycol, Macrogol 4000, Fortrans, Ipsen Pharma, France). Four hours later, mice received FMT by intragastric gavage (200 μl of resuspended feces prepared as described above). Mice were then allowed free access to food and water. FMT was repeated twice a week for two weeks before the induction of colitis and continued until the end of the protocol. Bowel cleansing was performed only on day 1.

To exclude the possibility that repeated force feeding of the intestinal microbiota had an effect *per se* on the phenotype, WTj mice were transplanted with their own fecal microbiota (WTj^WTj^) and used as the control. Additionally, we chose to use WT mice from Janvier and not cohoused mice to have a completely different microbiota as a baseline.

### Gavage with fungi and bacteria

*Candida tropicalis* ATCC 750 (ATCC, Molsheim, France) and *Malassezia restricta* CBS7877 (Westerdijk Institute, Utrecht, the Netherlands) were used in this study. *C. tropicalis* was grown on yeast extract peptone dextrose (YEPD) medium (BD Difco, Le Pont De Claix, France) for 24 h at 37 °C under agitation. *M. restricta* was grown statically on modified Dixon (mDixon) medium (BD Difco, Le Pont De Claix, France) for 72 h at 34 °C. The cultures were then washed twice in PBS, and a yeast suspension of 10^7^ CFU/mL in 200 μL of PBS or control medium (PBS) was administered daily to mice by intragastric gavage for 7 days before the induction of colitis, and this was continued until the end of the protocol.

*Lachnospiraceae* strains were also used in the study. *Marvinbryantia* and *Lachnospiraceae* bacterium WCA-9-b2 were isolated from the feces of D-1/2KO mice. Briefly, feces from 10 different female D-1/2KO mice were collected in a jar with Genebox Anaer® (Biomerieux, France) to maintain anaerobic conditions. Samples were transferred to an anaerobic chamber and resuspended in pre-reduced complete medium (37 g/L BHI (BD, Difco, USA) + Yeast extract (BD, Difco, USA) 1% + Hemin 5mg/ml (Sigma–Aldrich, St. Louis, MO, USA) + Maltose 0.1% (Sigma–Aldrich, St. Louis, MO, USA) + Cellobiose 0.1% (Sigma–Aldrich, St. Louis, MO, USA) + Cysteine 0.05% (Sigma–Aldrich, St. Louis, MO, USA) + Vitamin K1 0.0001% (Sigma–Aldrich, St. Louis, MO, USA) + Vitamin K3 3.2 μg/ml (Sigma–Aldrich, St. Louis, MO, USA)). They were then successively diluted up to 10^−7^, and 100 μl of each dilution was spread on agar complete media and incubate for 2 to 3 days at 37 °C in anaerobic conditions. Eighty strains from isolated colonies were sub-cultured in agar complete media and incubate for 2 to 6 days at 37 °C in anaerobic conditions. Then, strains were identified by 16S PCR sequencing.

*Blautia hansenii* ATCC 27752 was isolated from human feces and provided by ATCC. The bacteria were grown overnight on pre-reduced complete medium (see above) at 37 °C in a static incubator under anaerobic conditions, aliquoted at 5 × 10^8^ CFU/mL and stored at − 80 °C until use. Mice were fasted for 1 h and then subjected to bowel cleansing by oral-gastric gavage with PEG (Macrogol 4000, Fortrans, Ipsen Pharma, France). Four hours later, mice received a bacterial suspension of 10^8^ CFU in 200 μL of PBS or control medium. Bacterial gavage was administered daily to mice by intragastric gavage for 7 days before the induction of colitis, and this was continued until the end of the protocol. Bowel cleansing was only performed on day 1.

### Fluconazole treatment

For the antifungal treatment, fluconazole (0.5 mg/mL, Sigma–Aldrich, St. Louis, MO, USA) or control vehicle (PBS) was delivered to mice via drinking water for 7 days before the induction of colitis and continued until the end of the protocol.

### Short-chain Fatty Acid (SCFA) administration

Isobutyric acid (0.25 mol/L, Sigma–Aldrich, St. Louis, MO, USA), valeric acid (0.25 mol/L, Sigma–Aldrich, St. Louis, MO, USA), isovaleric acid (0.25 mol/L, Sigma–Aldrich, St. Louis, MO, USA), or control vehicle (PBS) was administered daily to mice by intragastric gavage for 7 days before the induction of colitis, and this was continued until the end of the protocol.

### Tissues and samples

Mice were euthanized by cervical dislocation. The distal colon was fixed in 4% paraformaldehyde (Electron Microscopy Sciences, Hatfield, PA, USA), and the proximal colon was flushed and frozen for further RNA extraction. Cecal contents were collected and frozen for SCFA quantification. Fecal samples were collected at day 0 and day 7 and at the end of the protocol (day 12) and frozen for gut microbiota analysis and fecal lipocalin level measurements. All samples were stored at − 80 °C until use.

### Histology

Colon samples for histological studies were maintained at 4 °C in 4% paraformaldehyde and then embedded in paraffin. Four-micrometer sections (three sections per sample) were stained with hematoxylin and eosin (H&E, Sigma–Aldrich, Saint Louis, USA) and then examined in a blinded manner using a BX43 Olympus microscope to determine the histological score according to previously described methods [12–14].

### SCFA analysis in cecal samples

Samples were water-extracted, and proteins were precipitated with phosphotungstic acid. A volume of 0.1 μl of the supernatant was analyzed for SCFAs on a gas–liquid chromatograph (Nelson 1020; Perkin-Elmer, St. Quentin en Yvelines, France) equipped with a split-splitless injector, a flame-ionization detector and a capillary column (15 m × 0.53 mm, 0.5 μm) impregnated with SP 1000 (FSCAP Nukol; Supelco, Saint-Quentin-Fallavier, France). The carrier gas (He) flow rate was 10 ml/min, and the inlet, column, and detector temperatures were 175, 100, and 280 °C, respectively. 2-Ethylbutyrate was used as the internal standard. Data were collected and peaks integrated using Turbochrom v6 software (Perkin Elmer, Courtaboeuf, France). Cecal SCFA concentrations are expressed as μmol/g of stool.

### Quantification of fecal lipocalin-2 (LCN2) levels

Frozen fecal samples were weighed and suspended in cold PBS. The samples were then agitated on a Precellys (Bertin Corp., France) for 40 s at 5000 rpm with 4.5-mm glass beads to obtain a homogenous fecal suspension. The samples were then centrifuged for 5 min at 10,000 × *g* (4 °C), and clear supernatants were collected and stored at − 20 °C until analysis. LCN2 levels were estimated using a DuoSet murine LCN2 ELISA kit (R&D Systems, Minneapolis, USA) according to the manufacturer’s instructions and expressed as ng/mg of stool.

### RNA extraction and gene expression analysis using quantitative real-time PCR (qRT–PCR)

Total RNA was isolated from colon samples using an RNeasy Mini Kit (Qiagen, Hilden, Germany), including a DNAse treatment step, according to the manufacturer’s instructions. Quantitative RT–PCR was performed using a Luna® Universal One-Step RT–qPCR Kit (New England Biolabs, Massachusetts, USA) followed by qPCR using Luna® Universal qPCR Master Mix (New England Biolabs, Massachusetts, USA) in a StepOnePlus apparatus (Applied Biosystems, Foster City, CA, USA) with specific mouse oligonucleotides. Amplification was initiated with an enzyme activation step at 95 °C for 10 min, followed by 40 cycles consisting of a 15 s denaturation step at 95 °C and a 60 s annealing step at 60 °C and a melting curve consisting of a step of temperature increase from 60 °C to 95 °C with a fluorescence analysis every 0.3 s. The primer sequences of the amplified target are listed in Supplemental Table 1. We used the 2^−ΔΔCt^ quantification method with mouse GAPDH as a control.

### Fecal DNA extraction

Fecal total DNA was extracted from weighed stool samples as previously described [15], with modifications. After nucleic acid precipitation with isopropanol, DNA suspensions were incubated overnight at 4 °C and centrifuged at 20,000 × *g* for 30 min. The supernatants were transferred to a new tube containing 2 μL of RNase (RNase A, 10 mg/ml; EN0531; Fermentas, Villebon sur Yvette, France) and incubated at 37 °C for 30 min. Nucleic acids were precipitated by the addition of 1 ml of absolute ethanol and 50 μL of 3 M sodium acetate and centrifuged at 20,000 × *g* for 10 min. The DNA pellets were washed with 70% ethanol 3 times, dried and resuspended in 100 μl of Tris-EDTA (TE) buffer (10 mM Tris-HCl, 1 mM EDTA, adjusted pH 8). The DNA suspensions were stored at − 20 °C for real-time qPCR analysis of the 16S rDNA or ITS2 sequences.

### Fungal and bacterial quantification via quantitative PCR (qPCR)

Fecal extracted DNA was subjected to qPCR by using Luna® Universal qPCR Master Mix (New England Biolabs, MA, USA) for quantification of all fungal sequences or by using TaqMan Gene Expression Assays (Life Technologies) for quantification of all bacterial sequences. For all fungal quantification, amplification was initiated with an enzyme activation step at 95 °C for 5 min, followed by 45 cycles consisting of a 15 s denaturation step at 94 °C, a 30 s annealing step at 55 °C, and a 30 s elongation step at 72 °C. This was followed by a single step of 5 min at 72 °C and a melting curve consisting of a step of temperature increase from 60 °C to 95 °C with a fluorescence analysis every 0.3 s. For all bacterial quantification, amplification was initiated with an enzyme activation step at 50 °C for 2 min and 95 °C for 10 min, followed by 40 cycles consisting of a 15 s denaturation step at 95 °C and a 60 s annealing/extension step at 60 °C. The probes and primers for the bacterial and fungal genes are listed in Supplemental Table 1. We used the 2^−ΔΔCt^ quantification method with fecal weight and calibrated the assay to the control group.

### 16S DNA gene and ITS2 sequencing

Bacterial diversity was determined for each sample by targeting a variable portion of the ribosomal genes. PCR was performed to prepare amplicons using V3-V4 oligonucleotides (PCR1F_460: 5′ CTTTCCCTACACGACGCTCTTCCGATCTACGGRAGGCAGCAG 3′, PCR1R_460: 5′ GGAGTTCAGACGTGTGCTCTTCCGATCTTACCAGGGTATCTAATCCT 3′). Amplicon quality was verified by gel electrophoresis, and the amplicons were sent to the @BRIDGe platform for sequencing on an Illumina MiSeq (Illumina, San Diego, CA, USA).

A similar approach was used for the fungal microbiota using the ITS2 primers 5′-GTGARTCATCGAATCTTT-3′ (sense) and 5′-GATATGCTTAAGTTCAGCGGGT-3′ (antisense) and the optimized and standardized ITS2 amplicon library preparation protocol (Metabiote, GenoScreen).

### 16S and ITS2 sequence analysis

For 16S sequences, the sequences were demultiplexed and quality filtered using the QIIME2 version 2021.2.0 software package [16]. The sequences were then assigned to OTUs using the UCLUST algorithm [17] with a 97% pairwise identity threshold and classified taxonomically using the Greengenes reference database (version 13.8) [18]. Rarefaction was performed on both datasets and used to compare the relative abundances of OTUs across samples. Alpha diversity was estimated using the Shannon diversity index or the number of observed species. Beta diversity was measured by a Jaccard distance matrix and was used to build principal coordinates analysis (PCoA) plots. The linear discriminant analysis (LDA) effect size (LEfSe) algorithm was used to identify taxa that were specific to a genotype [19].

For ITS2 sequences, data were processed using the FROGS pipeline (available from http://frogs.toulouse.inra.fr), established in Toulouse, France [20], for sequence quality control, filtering, and affiliation of taxa. The sequences were assigned to OTUs with a 97% threshold of pairwise identity and classified taxonomically using the UNITE ITS database (version 8_2) [21]. The phyloseq package for R was used for alpha and beta diversity analyses as well as all illustrations. The Deseq2 package for R was used for differential analysis of OTUs with respect to the different phenotypes [22]. The LEfSe algorithm was used to identify taxa that were specific to genotype.

Deposition of the raw sequence data in the SRA database from the NCBI, the accession numbers are the following: PRJNA824403 and PRJNA824345.

### Bone marrow-derived dendritic cell (BMDC) preparation and stimulation

Primary cultures of BMDCs were obtained from WT, D-1KO, D-2KO, and D-1/2KO mice as previously described [23] with minor modifications. Briefly, bone marrow cells isolated from mouse tibiae and femurs were washed with Roswell Park Memorial Institute (RPMI) 1640 and passed through 40 μm cell filters (Falcon, USA). Cells were then cultured and differentiated in petri dishes in 10 mL of RPMI 1640 with 10% (vol/vol) FCS (Eurobio Scientific), 100 U/mL penicillin, 100 μg/mL streptomycin (Sigma–Aldrich) and 20 ng/mL murine granulocyte-macrophage colony-stimulating factor (GM-CSF Peprotech, Germany) for 9 days at 37 °C and 5% CO_2_.

On day 9, immature BMDCs were harvested and seeded into 96-well plates at 10^5^ cells/well. The cells were stimulated with bacterial strains (1:5 cell-to-bacteria ratio) killed by UV, lipopolysaccharide (LPS) (150 ng/mL), depleted zymosan (100 μg/mL, InvivoGen), and yeast mannan (100 μg/mL, Sigma–Aldrich) overnight at 37 °C. The culture supernatants were collected, and the levels of IL-6 and TNFα were measured by ELISA kits (Thermo Fisher Scientific) according to the manufacturer’s protocol.

### Statistical analysis

GraphPad Prism version 7 (San Diego, CA, USA) was used for all analyses and preparation of graphs. For all data displayed in graphs, the results are expressed as the mean ± SEM (*n* = 4 to 16 per group). For comparisons between two groups, a two-tailed Student’s *t* test for unpaired data or a nonparametric Mann–Whitney test was used. For comparisons among more than two groups, one-way analysis of variance (ANOVA) and a post hoc Tukey test or a nonparametric Kruskal–Wallis test followed by a post hoc Dunn’s test were used. For comparisons with multiple factors, two-way ANOVA and a post hoc Tukey test were used. For all statistical tests, differences with a *p* value less than 0.05 were considered to be statistically significant: *p < 0.05, **p < 0.01, ***p < 0.001.

## Results

### Double Dectin-1/2 deficiency ameliorates DSS-induced colitis

**Fig. 1:**
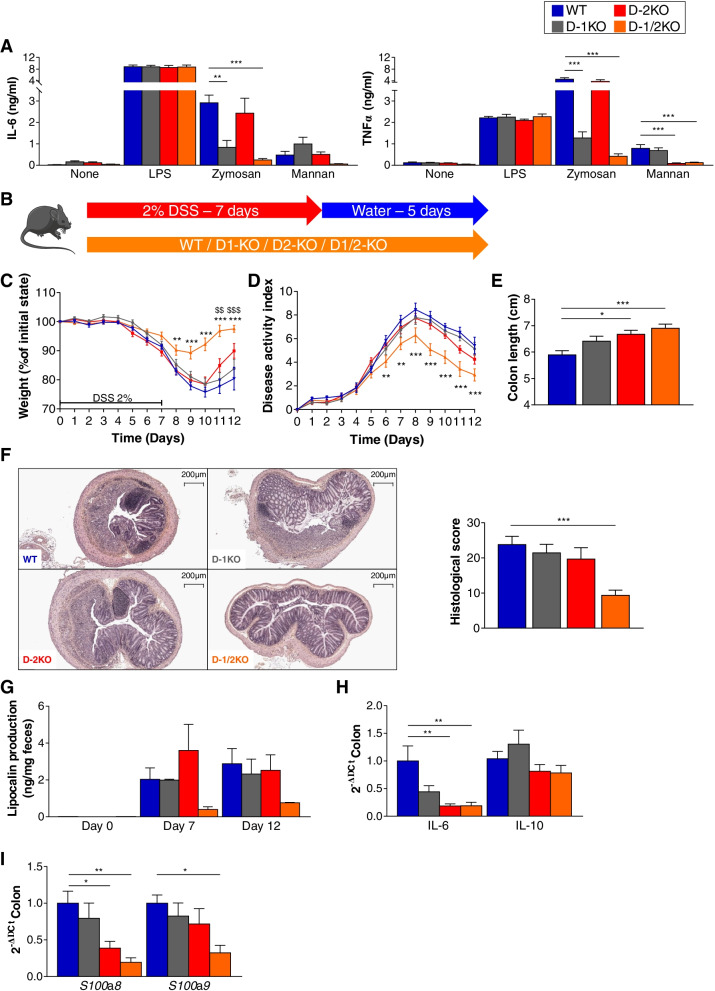
Dectin-deficient mice react differently to DSS-induced colitis. **A** ELISA of the expression of IL-6 (left panel) and TNFα (right panel) in BMDC cultures from wild-type (WT), Dectin-1-deficient (D-1KO), Dectin-2-deficient (D-2KO), and Dectin-1/2-deficient (D-1/2KO) mice stimulated with fungal ligands (zymosan and mannan). **B**–**F** WT, D-1KO, D-2KO, and D-1/2KO mice received dextran sulfate sodium (DSS) for 7 days. WT *n* = 13, D-1KO *n* = 15, D-2KO *n* = 14, D-1/2KO *n* = 15. **B** Experimental design for the administration of DSS. **C** Weight of DSS-exposed mice. **D** Disease activity index (DAI) of DSS-exposed mice. **E** Length of the colons of mice treated with DSS. **F** Representative H&E-stained images of proximal colon cross sections on day 12 after initial DSS exposure (left panel) and histological scores on day 12 (right panel). **G** Intestinal inflammation, expressed as the lipocalin levels in feces at day 0, day 7, and day 12. **H** Intestinal cytokines in the colon (qPCR). **I** Antimicrobial peptides expressed in the colon (qPCR). For statistical comparisons, (*) indicates D-1/2KO versus WT, and ($) indicates D-2KO versus WT. *p < 0.05, **^,$$^*p* < 0.01, ***^,$$$^*p* < 0.001

C57BL/6 D-1KO and D-2KO mice were collected from collaborators and bred in our animal facility for the production of littermates of the 4 following genotypes: Dectin-1-deficient mice (D-1KO), Dectin-2-deficient mice (D-2KO), mice double deficient for Dectin-1 and Dectin-2 (D-1/2KO), and control mice (wild-type, WT). The genotype of each mouse was verified by PCR on DNA extracted from mouse tissue samples. Additional confirmation of the genotype was obtained by comparing bone marrow-derived dendritic cell (BMDC) responses to the Dectin-1- and Dectin-2-specific fungal ligands zymosan and mannan, respectively. BMDC cultures from WT, D-1KO, D-2KO, and D-1/2KO mice were stimulated with zymosan and mannan, and culture supernatants were used for IL-6 and TNFα cytokine production quantification (Fig. 1A). As expected, D-1KO and D-1/2KO mice showed weak to no response to zymosan, while D-2KO and D-1/2KO mice did not recognize mannan ligands.

Using these 4 genotypes, we first investigated the role of Dectin-1 and Dectin-2 in dextran sodium sulfate (DSS)-induced colitis (Fig. 1B) by administering 2% DSS to D-1KO, D-2KO, D-1/2KO, and WT mice. No significant differences were observed between WT and D-1KO or D-2KO mice (Fig. 1D), although D-2KO mice seemed to exhibit better recovery than WT mice (Fig. 1C, E). However, D-1/2KO mice exhibited a strongly decreased severity of colitis from day 6, characterized by significantly lower weight loss (Fig. 1C) and disease activity index (DAI) (Fig. 1D). On day 12, after 5 days of recovery, D-1/2KO mice had a longer colon, characteristic of a better tissue condition (Fig. 1E) and a significantly lower histology score (Fig. 1F).

Quantification of lipocalin, a fecal marker of intestinal inflammation, showed a reduced level at day 7 and day 12 in D-1/2KO mice compared to WT mice, although the difference was not statistically significant (Fig. 1G). Similarly, the colonic expression of the proinflammatory cytokine IL-6 was decreased in D-2KO and D-1/2KO mice, while the expression of the anti-inflammatory cytokine IL-10 was unchanged (Fig. 1H). Based on previous work published on Dectin-1KO mice, we quantified the expression of two antimicrobial peptides (AMPs), *S100*a*8* and *S100*a*9*, in the colon of WT and KO mice and showed that the expression level was significantly reduced in the double-KO mice at day 12 for both markers and that the reduction was significant for only *S100*a*8* in D-2KO mice (Fig. 1I), suggesting reduced inflammation and potential effects on the gut microbiota composition. However, these differences were not visible at baseline before DSS challenge (Supp. Fig. 1A). Altogether, these data confirmed the strong modification of the local host environment in D-1/2KO mice compared to WT mice in the colitis setting, with a lower global inflammatory status in the double KO mice throughout inflammation.

### The microbiota of D-1/2KO mice is associated with DSS-induced colitis protection

**Fig. 2:**
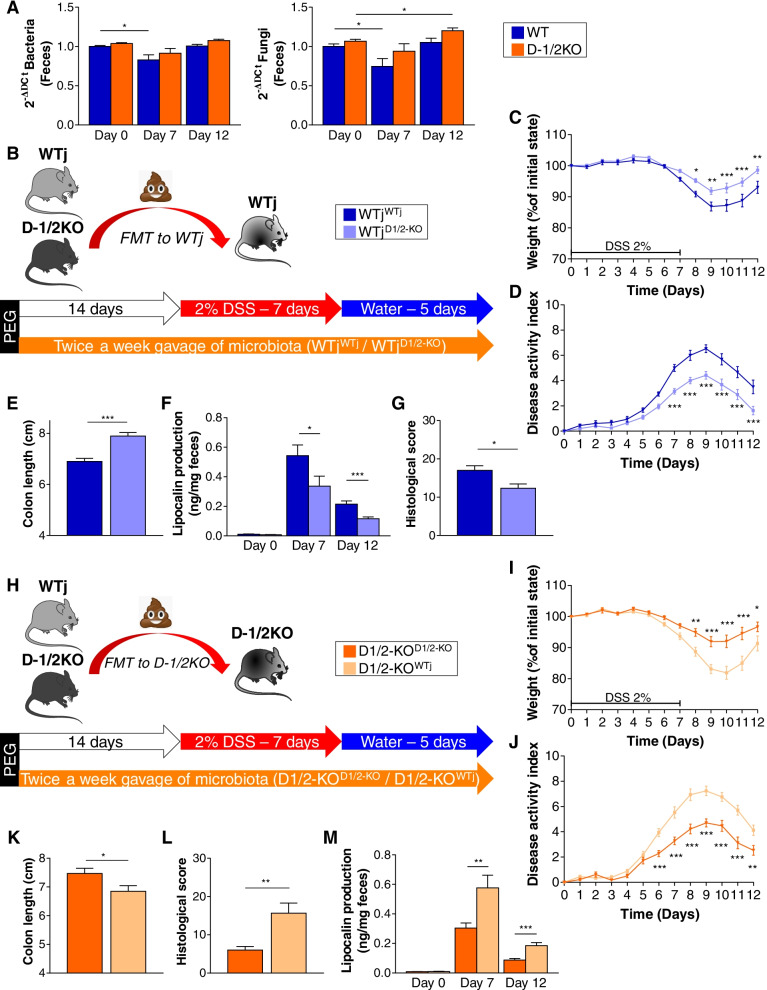
The fecal microbiota from double Dectin-deficient mice ameliorates DSS-induced colitis. **A** Wild-type (WT) and Dectin-1/2-deficient (D-1/2KO) mice received dextran sulfate sodium (DSS) for 7 days. WT *n* = 13, D-1/2KO *n* = 15. **A** Bacterial (left panel) and fungal (right panel) quantities in feces at day 0, day 7, and day 12, determined by qPCR. **B**–**G** Wild-type mice from Janvier Laboratory transplanted with their own intestinal microbiota (WTj^WTj^) or transplanted with the intestinal microbiota of Dectin-1/2-deficient mice (WTj^D-1/2KO^) received DSS for 7 days. WTj^WTj^ *n* = 18, WTj^D-1/2KO^ *n* = 20. **H**–**M** Dectin-1/2-deficient mice transplanted with their own intestinal microbiota (D-1/2KO^D-1/2KO^) or transplanted with the intestinal microbiota of wild-type mice from Janvier Laboratory (D-1/2KO^WTj^) received DSS for 7 days. D-1/2KO^D-1/2KO^ *n* = 17, D-1/2KO^WTj^ *n* = 18. **B**/**H** Experimental design for fecal microbiota transplantation and administration of DSS. **C**/**I** Weight of DSS-exposed mice. **D**/**J** Disease activity index (DAI) of DSS-exposed mice. **E**/**K** Length of the colons of mice treated with DSS. **F**/**L** Histological scores on day 12. **G**/**M** Intestinal inflammation, expressed as the lipocalin levels in feces at day 0, day 7, and day 12. *p < 0.05, **p < 0.01, ***p < 0.001

Modification of the host immune response directly influences the microbiota since the equilibrium between microbiota and host cells is a subtle balance mainly regulated by the innate response pathways. The principal effectors are the AMPs produced by the host cells. We thus investigated the effect of combined Dectin-1 and Dectin-2 deficiency on global bacterial and fungal loads at different time points. We first observed a global effect of DSS treatment on the WT microbiota after 7 days, before the recovery phase, with reduced abundances of both the bacterial and fungal populations. The two populations returned to normal after recovery on Day 12. However, D-1/2KO exhibited no modification of the bacterial abundance even at Day 7, while a significant increase in the fungal load was observed at the end (Day 12) of DSS-induced colitis (Fig. 2A).

Both the bacterial and fungal intestinal microbiotas have been identified as important players in IBD. We considered that the absence of both Dectin-1 and Dectin-2 receptors could be associated with specific alterations in the intestinal microbiota that may result in the protection of D-1/2KO mice. To test the potential involvement of the microbiota in the phenotype of D-1/2KO mice, we transplanted the fecal microbiota of wild-type mice from Janvier Laboratory (WTj) or D-1/2KO mice into conventional WTj mice and induced colitis via DSS treatment (Fig. 2B). Compared to WTj mice receiving the WTj mouse microbiota (WTj^WTj^), those receiving the D-1/2KO mouse microbiota (WTj^D-1/2KO^) showed significantly reduced colitis severity, as indicated by their reduced body weight loss (Fig. 2C) and DAI (Fig. 2D), increased colon length (Fig. 2E), reduced levels of fecal lipocalin (Fig. 2F) and histological score (Fig. 2G and Supp. Fig. 2A).

We then assessed the role of the genotype on the protection by testing whether transplanting the wild-type microbiota into D-1/2KO mice would revert the protection. With this aim, we transplanted the intestinal microbiota of conventional mice into D-1/2KO mice before induction of colitis with DSS (Fig. 2H). D-1/2KO mice receiving the WTj mouse microbiota (D-1/2KO^WTj^) showed increased severity of colitis at Day 8, characterized by significantly stronger weight loss (Fig. 2I), a higher DAI (Fig. 2J), colon shortening (Fig. 2K), histological damage (Fig. 2L and Supp. Fig. 2B), and increased lipocalin levels (Fig. 2M), compared to the control mice ^D-12KO^ transplanted with their own fecal microbiota (D-1/2KO^D-1/2KO^).

These two experiments demonstrate that the protection of D-1/2KO mice against DSS-induced colitis was largely due to the intestinal microbiota.

### The fungal microbiota is not involved in D-1/2KO mouse protection

**Fig. 3:**
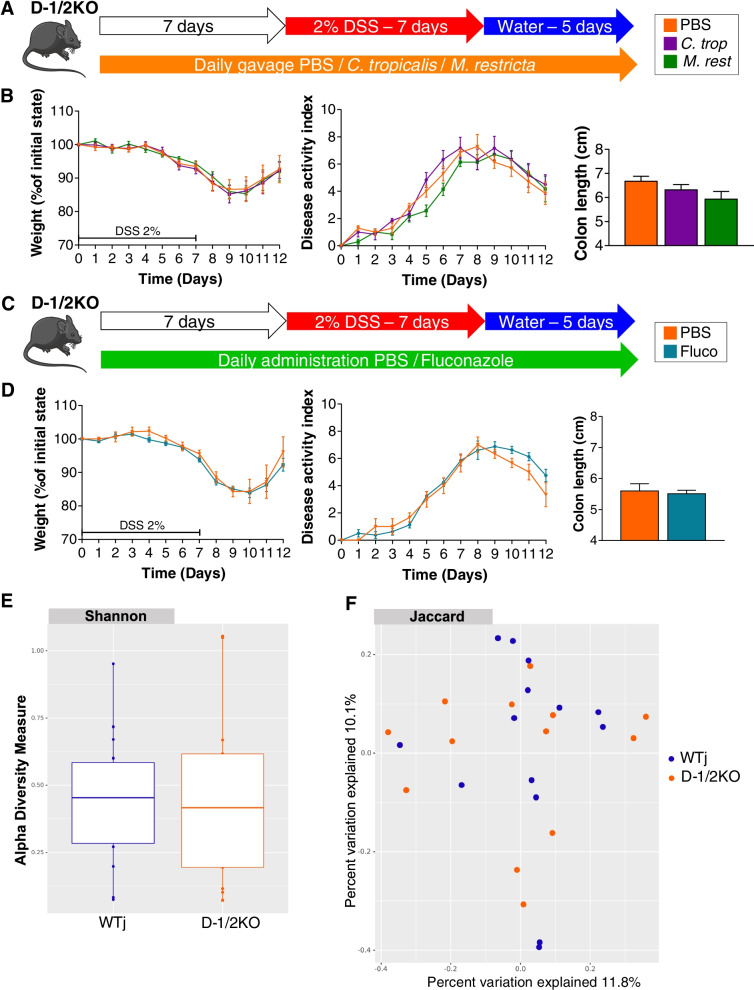
Fungi do not seem to have an effect on double Dectin-deficient mice with DSS-induced colitis. **A**, **B** Dectin-1/2-deficient (D-1/2KO) mice received *C. tropicalis* (*C. trop*) or *M. restricta* (*M. rest*) and then dextran sulfate sodium (DSS) for 7 days. PBS *n* = 7, *C. trop n* = 6, *M. rest n* = 7. **A** Experimental design for the administration of fungi and DSS. **B** Weight of DSS-exposed mice (left panel), disease activity index (DAI) of DSS-exposed mice (middle panel) and length of the colons of mice treated with DSS (right panel). **C**, **D** D-1/2KO mice received fluconazole (Fluco) and then DSS for 7 days. PBS *n* = 3, Fluco *n* = 8. **C** Experimental design for the administration of an antifungal agent and DSS. **D** Weight of DSS-exposed mice (left panel), disease activity index (DAI) of DSS-exposed mice (middle panel) and length of the colons of mice treated with DSS (right panel). **E**, **F** Wild-type mice from Janvier Laboratory (WTj) and Dectin-1/2 deficient (D-1/2KO) mice without challenge. WTj *n* = 14, D-1/2KO *n* = 13. **E** Shannon index describing the alpha diversity of the fungal microbiota (ITS) in the fecal microbiota. **F** Beta diversity. Principal coordinate analysis of Jaccard distance, with each sample colored according to the genotype

Our FMT data showed that the protection of D-1/2KO mice was due to an altered intestinal microbiota. As Dectin-1 and Dectin-2 are known for their role in fungal recognition, we evaluated how modification of the fungal population would affect DSS-induced susceptibility. With this aim, we used two commensal fungi, *Candida tropicalis* and *Malassezia restricta*, described for their potential proinflammatory effect on a DSS-induced colitis model [7, 24] (Fig. 3A). Surprisingly, daily gavage of D-1/2KO mice with both fungi 7 days before and during DSS treatment had no effect on the different macroscopic markers, weight loss, DAI or colon length (Fig. 3B).

In our first experiment (Fig. 2A), fecal analysis in D-1/2KO mice showed an increase in fungal abundance, so we reasoned that the phenotype observed in D-1/2KO mice could be due to the modification of the fungal microbiota load. To test this hypothesis, we treated D-1/2KO mice with a broad-spectrum antifungal drug (fluconazole) and characterized the effect of this treatment on DSS-induced colitis (Fig. 3C). Again, as illustrated in Fig. 3D, modification of the fungal population did not affect the susceptibility of D-1/2KO mice to colitis. These data suggest that even though Dectin-1 and Dectin-2 are two fungal receptors, their effects on the susceptibility of D-1/2KO mice to DSS-induced colitis did not seem to be linked to an effect on the fungal population.

To further characterize the fungal microbiota in mice deficient in both Dectin-1 and Dectin-2, we investigated the effect of double Dectin deficiency on the fungal microbiota composition by amplicon-based sequence analysis targeting ITS2. We analyzed the mycobiota of D-1/2KO and WTj mice at baseline. Surprisingly, the analysis showed that D-1/2 deficiency did not induce global or specific fungal dysbiosis, since no difference between groups based on alpha diversity (Fig. 3E) or beta diversity (Fig. 3F) was observed. Testing for the identification of specific fungi that would affect the phenotype by bioinformatic differential abundance analysis using different statistical tools did not give any significant results (data not shown), confirming the absence of modification of the fungal microbiota in D-1/2KO mice.

### Lack of Dectin-1 and Dectin-2 is associated with an altered bacterial microbiota

**Fig. 4:**
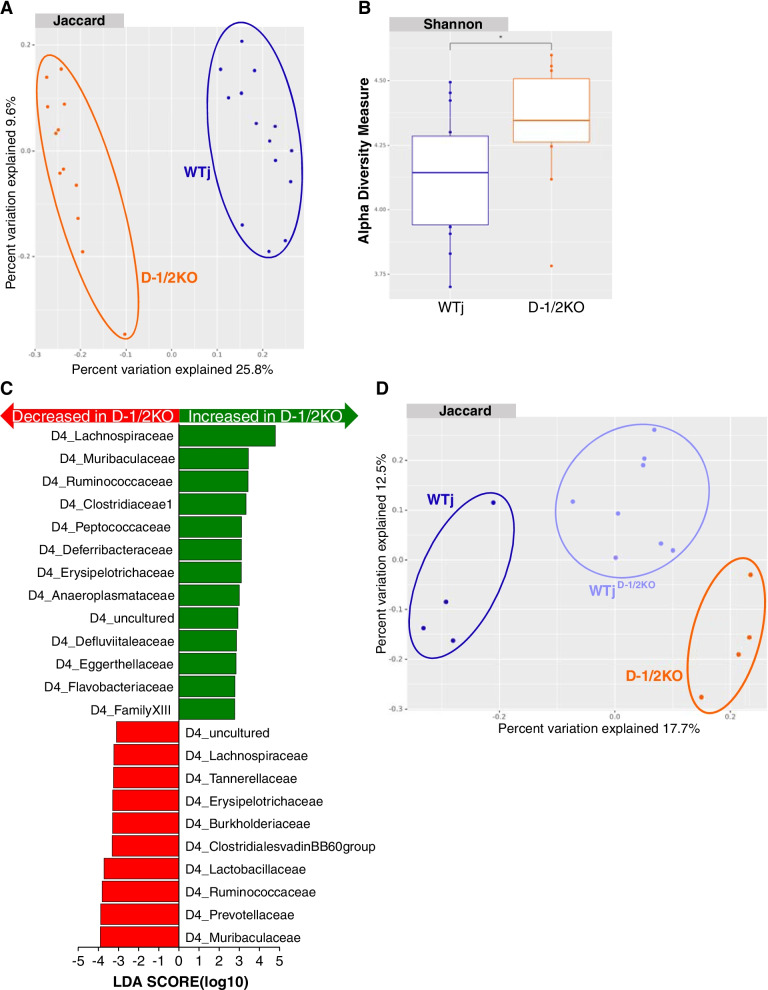
The bacterial microbiota plays an important role in double Dectin-deficient mouse protection. **A**–**C** Wild-type mice from Janvier Laboratory (WTj) and Dectin-1/2 deficient (D-1/2KO) mice without challenge. WTj *n* = 14, D-1/2KO *n* = 12. **A** Beta diversity. Principal coordinate analysis of Jaccard distance, with each sample colored according to the genotype. **B** Shannon index describing the alpha diversity of the bacterial microbiota (16S) in the fecal microbiota. **C** Taxa with the largest differences (LDA > 2) in abundance by linear discriminant analysis (LEfSe) (LDA > 2). **D** WTj, D-1/2KO and WTj transplanted with the intestinal microbiota of Dectin-1/2-deficient mice (WTj^D-1/2KO^) mice without challenge. WTj *n* = 4, D-1/2KO *n* = 4, WTj^D-1/2KO^ *n* = 8. **D** Beta diversity. Principal coordinate analysis of Jaccard distance, with each sample colored according to the genotype

Since the microbiota seems to be central to gut protection in D-1/2KO mice (see FMT treatment), after we demonstrated that the fungal microbiota was not essential for the protective phenotype, we investigated whether the bacterial microbiota differed between these mice and the control. Fecal 16S sequencing and analysis showed that the bacterial microbiota in D-1/2KO mice clustered significantly differently than the gut microbiota from WTj mice (Fig. 4A). Alpha diversity analysis with the Shannon index also illustrated significant global modification of the bacterial microbiota (Fig. 4B). To identify whether particular bacteria were responsible for this dysbiosis and potentially involved in the protection against gut inflammation, we performed a differential abundance analysis using several independent tools. As illustrated in Fig. 4C, linear discriminant analysis (LEfSe) showed significant differences in several taxonomic ranks. One of the stronger signals was the increase in *Blautia* sp*.* and more generally in bacteria from the *Lachnospiraceae* family in D-1/2KO mice compared to WTj mice. Additional comparison of the D-1/2KO gut microbiota to the gut microbiota of their WT littermates also showed comparable results with *Lachnospiraceae* enrichment (data not shown).

As illustrated in Fig. 4D, the bacterial microbiota of WTj^D-1/2KO^ mice was modified and was intermediate between the microbiota of the donor D-1/2KO mice and recipient WTj mice, suggesting partial implantation of the donor microbiota that was sufficient for protection against gut inflammation.

### *Lachnospiraceae* strains play a role in D-1/2KO resistance against gut inflammation

Based on the data reported above, we next explored the biological impact of the increased abundance of the *Lachnospiraceae* family in D-1/2KO mice.

As the *Lachnospiraceae* family is known to produce short-chain fatty acids (SCFAs) [25], we quantified cecal SCFA production at baseline in D-1/2KO and WTj mice. No differences were observed between D-1/2KO mice and WTj mice for the three dominant SCFAs (butyrate, acetate and propionate) (Supp Fig. 3A left panel). However, the levels of branched SCFAs (isobutyrate, valerate, and isovalerate), a class of fatty acids present at much lower concentrations in the feces, were significantly increased in D-1/2KO mice compared to the control group (Supp Fig. 3A right panel). To determine whether these branched SCFAs could protect against DSS-induced colitis, we administered isobutyric acid, valeric acid, and isovaleric acid to WTj mice (Supp Fig. 3B). None of these acids improved colitis severity (Supp Fig. 3C-E), even though isovaleric acid-treated mice showed a significantly lower DAI from day 9 (Supp Fig. 3D).

As a single metabolite might not be responsible for the complete phenotype, we reasoned that using *Lachnospiraceae* isolated from the feces of D-1/2KO mice might allow the identification of potential probiotic strains. Fecal samples from D-1/2KO mice enriched in *Lachnospiraceae* were used for several rounds of isolation of *Lachnospiraceae* bacteria for subsequent in vitro and in vivo experiments. The need for strict anaerobic conditions and a complex medium composition hampered the selection of a large number of isolates. However, we were able to isolate 5 different bacteria from the *Lachnospiraceae* family. The *Blautia* genus was the strongest hit identified by our LEfSe analysis (Fig. 4C), but we were unable to isolate any *Blautia* strains from the feces of D-1/2KO mice. Thus, for the purpose of our analysis, we used a *Blautia* strain from an international collection: *Blautia hansenii* ATCC 27752.

**Fig. 5:**
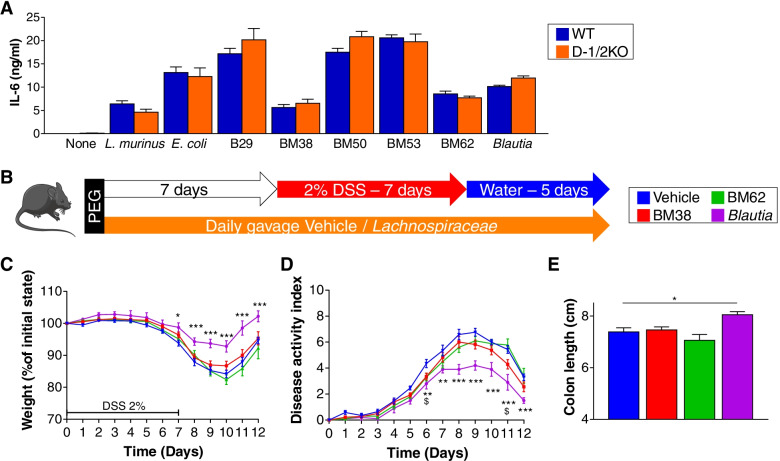
*Lachnospiraceae* strains can protect mice from DSS-induced colitis. **A** ELISA of the expression of IL-6 in BMDC cultures from wild-type (WT) and Dectin-1/2-deficient (D-1/2KO) mice stimulated with dead bacteria. **B**–**E** Mice received several *Lachnospiraceae* (BM38, BM62, or *Blautia*) or medium (vehicle) and dextran sulfate sodium (DSS) for 7 days. Vehicle *n* = 17, BM38 *n* = 18, BM62 *n* = 10, *Blautia n* = 10. **B** Experimental design for the administration of *Lachnospiraceae* and DSS. **C** Weight of DSS-exposed mice. **D** Disease activity index (DAI) of DSS-exposed mice. **E** Length of the colons of mice treated with DSS. For statistical comparisons, (*) indicates *Blautia* versus vehicle, and ($) indicates BM38 versus vehicle. *^,$^*p* < 0.05, **p < 0.01, ***p < 0.001

Before in vivo experiments, we used in vitro screening of BMDCs to select the best strains with potential anti-inflammatory effects. From this screen, we selected the strains BM38, BM62 and *B. hansenii* ATCC 27752 for in vivo experiments (Fig. 5A and Supp Fig. 4A) since these strains elicited lower IL-6 and TNF alpha (for BM38 only) production after coincubation with BMDCs.

BM38 and BM62 isolates were identified using 16S sequence blast against the NCBI database. BM38 was identified as *Marvinbryantia* sp*.* and BM62 as *Sporofaciens musculi* (novel genus and species, formerly called *Lachnospiraceae* bacterium WCA-9-b2).

To test whether these strains might have a protective effect during DSS-induced colitis, we gavaged the 3 *Lachnospiraceae* strains to WTj mice prior to DSS-induced colitis (Fig. 5B). After DSS treatment and recovery, we identified no potential protective role for *Marvinbryantia* sp*.* or *Sporofaciens musculi* (BM38 and BM62, respectively). However, the *B. hansenii* ATCC 27752 strain reduced the severity of colitis from Day 6, characterized by significantly lower weight loss (Fig. 5C), a lower DAI (Fig. 5D) and reduced colon shortening (Fig. 5E).

Overall, these results suggest that the lack of Dectin-1 and Dectin-2 in mice with DSS-induced colitis is associated with a specific bacterial microbiota modification, resulting in the protection of D-1/2KO mice. The *Lachnospiraceae* family seems to play a role in this protection.

## Discussion

The human gut microbiota has been the subject of in-depth analysis over the past decade, identifying this community of microorganisms as a new actor in health and (in many) diseases, notably IBD. While the role of the bacterial gut microbiota in IBD has been intensively studied, the gut fungal microbiota (mycobiota) is now also recognized for its role in gut inflammation [26]. Indeed, previous works from our group and D.M. Underhill’s team showed that mice defective for genes involved in innate immunity toward fungi (Dectin-1 and Card9) are more susceptible to intestinal inflammation, highlighting the potential role of fungal receptors in IBD [3, 4]. Since then, many studies have been initiated to decipher the crosstalk between the host and the fungal microbiota through the study of the complex network of fungal receptors and the associated signaling pathways. Among the receptors that detect fungal ligands, C-type lectin receptors (Dectin-1, Dectin-2, Dectin-3 and Mincle) are the most widely studied since they were initially linked to the immune response against fungal infections.

In this work, we studied Dectin-1KO, Dectin-2KO and double Dectin-1KO/Dectin-2KO mice and, in particular, the effects of the knockouts on the susceptibility to gut inflammation. We demonstrated that while Dectin-1KO mice were not impacted and Dectin-2KO mice had a slightly modified course of colitis compared to WT mice, the double Dectin-1KO and Dectin-2KO mice were strongly protected against DSS-induced colitis. Interestingly, we showed that this protection was due to a modified bacterial, rather than fungal, microbiota.

While a large amount of data is available on the impact of Dectin-1 on the gut microbiota and host health, very little is known about the other receptors, especially Dectin-2, for which, to our knowledge, no data are available in the literature. Additionally, interactions have been shown among CLRs: between Dectin and Mincle but also between Dectin receptors. For instance, interactions between Dectin-2 and Dectin-3 have been reported to be involved in the recognition of *Candida albicans* in vitro [27]. While Dectin-2 and Dectin-3 seem to physically interact at the cell surface to trigger the signaling cascade, Dectin-2 and Dectin-1 either cooperate or have redundant functions for global immune signaling. For instance, Dectin-1 was found to functionally collaborate with Dectin-2 to induce an optimal Th17 response to *C. albicans* [28]. Furthermore, a recent study showed that stimulation of human monocyte-derived dendritic cells (DCs) or macrophages with human thioredoxin resulted in the production of IL-1β and IL-23, while it did not do so in either Dectin-1- or Dectin-2-deficient DCs, suggesting that both Dectin-1 and Dectin-2 have an effect on the secretion of IL-1β [29]. Apart from the synergistic effect, each dectin receptor can function as a rescue alternative in the absence of the other. Indeed, a recent study showed that in the presence of Dectin-2, *Histoplasma capsulatum* could induce NLRP3 inflammasome activation and IL-1β production via Dectin-2 but not Dectin-1. However, in the absence of Dectin-2, Dectin-1 induced IL-1β production, although to a lower degree than Dectin-2 [30]. Altogether, these data suggest that following the phenotype of the double mutant would give particularly interesting insight into the global role of these CLRs in the crosstalk between the host and gut microbiota. Thus, we initiated our study by investigating wild-type (WT), Dectin-1KO (D-1KO), Dectin-2KO (D-2KO), and Dectin-1KO/Dectin-2KO (D-1/2KO) mice. To date, only one publication is available on D-1/2KO mice with *H. capsulatum* infection [30]; consequently, this study is the first to describe the D-1/2KO mouse phenotype in a model of gut inflammation.

Strikingly, D-1/2KO mice were robustly and significantly protected against gut inflammation in this colitis model. Markers related to the gut microbiota also highlighted evident changes in D-1/2KO mice. First, unsurprisingly, the abundance of fungi was higher in D-1/2KO mice, and as both Dectin-1 and Dectin-2 are receptors for fungi, the regulation of the fungal load was less stringent in their absence. We also confirmed the lower immune response of D-1/2KO immune cells interacting with beta-glucan and mannan in an in vitro assay. On the other hand, bacterial abundance quantification showed no clear modification, while the concentration of an AMP (*S100*a*8*/*9*) was significantly decreased, suggesting broader modification of the gut microbiota, i.e., not limited to the fungal population. S100a8/9, the two components of calprotectin, a protein present at very high concentrations in the neutrophil cytoplasm, are also considered good markers of gut inflammation in humans and possibly new diagnostic markers [31]. However, their role in microbiota composition regulation has been recently highlighted in the modulation of the neonatal microbiota, as these proteins are present at high concentrations in maternal milk [32]. Consequently, low secretion of S100a8/9 in D-1/2KO mice could be a sign of low inflammation but could also affect the microbiota itself in a vicious or virtuous cycle.

We thus reasoned that the microbiota might be modified in D-1/2KO mice. A way to test causality for the role of the microbiota in the phenotype was to perform fecal material transfer experiments. Interestingly, the transfer of the D-1/2KO microbiota into completely independent mice was enough to transfer the complete protective phenotype, with all markers showing clear effects on the host during gut inflammation. We confirmed that the microbiota was the element changing the susceptibility to inflammation by replacing the D-1/2KO microbiota in D-1/2KO mice with a wild-type microbiota and demonstrated that the mice were as sensitive as wild-type mice. These results showed that the role of the genotype during the inflammation procedure was negligible but that all the protecting elements were carried by the bacterial microbiota itself. Remarkably, this was not the case in the study of Dectin-1KO alone performed by Iliev et al.*,* where the transfer itself was not enough to transfer the deleterious phenotype, while it was the case in the work of Tang and coworkers, where the transfer of the microbiota also transferred protection [3, 7]. This protection was driven by a modified bacterial microbiota enriched with *Lactobacillus murinus* in this case [3].

As Dectin-1 and Dectin-2 were initially described as receptors for fungal ligands and regulators of the fungal population, we hypothesized that the fungal population was most likely responsible for the resistance phenotype. To test this hypothesis, two experiments were set up, either by adding fungal pro-inflammatory strains (*C. tropicalis* or *M. restricta*), as was done in related studies by Tang et al. or Limon et al. [7, 24], or by completely modifying the fungal population by treatment with an antifungal drug (fluconazole). To our surprise, neither of the two experimental treatments changed the resistance phenotype in D-1/2KO mice, suggesting that if the fungal population was impacting the phenotype, it was in a very indirect manner. Our analyses of the gut mycobiota confirmed that there was no modification in the quality of the mycobiota.

However, a very strong modification of the bacterial microbiota was detected. Detailed analysis of the differences between the two microbiotas showed enrichment of several bacterial strains, including bacteria from the *Lachnospiraceae* family, such as *Blautia* sp., but not *Lactobacillacaeae*. We used fecal samples from D-1/2KO mice to isolate bacterial strains from the *Lachnospiraceae* family to test a possible protective effect. Of the several strains tested, only *Blautia hansenii* strains that came from our laboratory collection, but not mouse feces, were able to recapitulate the protective phenotype.

## Conclusions

Our data suggest that deletion of both major dectin receptors, Dectin-1 and Dectin-2, greatly influenced the susceptibility to DSS-induced colitis. Although Dectin receptors are mainly known to recognize fungal epitopes, the double deletion did not affect the fungal microbiota but strongly impacted its counterpart, the bacterial microbiota, a result previously observed by Tang et al. This modified bacterial microbiota is the causative agent of DSS resistance, and the effect could be attributed to the enrichment of *Lachnospiraceae*, such as *Blautia hansenii* strains. In the literature, interest in the potential effect of *Blautia* sp*.* is slowly growing. A recent review gathered the current knowledge on this growing genus that was joined by former *Ruminoccocus* and *Clostridium* genera [33]. As several *Blautia* sp*.* have been associated with metabolic and inflammatory diseases, the question of its potential probiotic role remains unanswered, although in the literature, a direct role between *Blautia* abundance and amelioration of health status has not yet been proven. This study is the first to confirm a potential direct effect of one *Blautia* sp*.* in protecting against gut inflammation. Further studies are needed to explore the mechanisms involved in this protection. In addition, we do not yet know the mechanism by which deletion of Dectin-1 and Dectin-2 strongly influences the bacterial microbiota. The hypothesized indirect effect of the fungal population on the bacterial community seems unlikely based on our current results. The more plausible hypothesis would be modification of the broad spectrum innate response that would affect mostly the bacteria, suggesting direct recognition of bacterial ligands by Dectin-1 and Dectin-2 or more complex indirect effects on the innate response equilibrium.

## Supplementary Information

**Additional file 1: Supplemental Fig. 1.** Antimicrobial peptides at baseline were not different. A. Antimicrobial peptides expressed in the colon of wild-type (WT) and Dectin-1/2-deficient (D-1/2KO) mice without challenge (qPCR). WT *n* = 8, D-1/2KO *n*= 9. **Supplemental Fig. 2.** Fecal microbiota transplantation either ameliorates or worsens DSS-induced colitis. (A) Wild-type mice from Janvier Laboratory transplanted with their own intestinal microbiota (WTj^WTj^) or transplanted with the intestinal microbiota of Dectin-1/2-deficient mice (WTj^D-1/2KO^) received dextran sulfate sodium (DSS) for 7 days. WTj^WTj^ *n* = 18, WTj^D-1/2KO^ *n* = 20. (B) Dectin-1/2-deficient mice transplanted with their own intestinal microbiota (D-1/2KO^D-1/2KO^) or transplanted with the intestinal microbiota of wild-type mice from Janvier Laboratory (D-1/2KO^WTj^) received dextran sulfate sodium (DSS) for 7 days. D-1/2KO^D-1/2KO^ *n* = 17, D-1/2KO^WTj^ *n* = 18. A-B. Representative H&E-stained images of proximal colon cross sections on Day 12 after initial DSS exposure. **Supplemental Fig. 3.** SCFA quantification and administration. A. Quantification of short-chain fatty acids (SCFAs) in the cecum of wild-type (WT) and Dectin-1/2-deficient (D-1/2KO) mice without challenge. WT *n* = 10, D-1/2KO *n*= 10. (B-E) Mice received several SCFAs (isobutyric acid, valeric acid or isovaleric acid) or vehicle (PBS) and dextran sulfate sodium (DSS) for 7 days. PBS *n* = 17, isobutyric acid *n* = 17, valeric acid *n* = 11, isovaleric acid *n* = 16. B. Experimental design for the administration of SCFAs and DSS. C. Weight of DSS-exposed mice. D. Disease activity index (DAI) of DSS-exposed mice. E. Length of the colons of mice treated with DSS. For statistical comparisons, (*) indicates isovaleric acid versus PBS, and ($) indicates isobutyric acid versus PBS. *^,$^*p* <0.05, **p <0.01. **Supplemental Fig. 4.** *Lachnospiraceae* strains co-culture with BMDC elicit different TNF-α production. A. ELISA of the expression of TNF-α in BMDC cultures from wild-type (WT) and Dectin-1/2-deficient (D-1/2KO) mice stimulated with dead bacteria.

**Additional file 2.**

## Data Availability

All data generated or analyzed during this study are included in this published article (and its supplementary information files). Deposition of the raw sequence data in the European Nucleotide Archive is in process; the accession number is pending.

## Abbreviations

AMP: Antimicrobial peptide
ANOVA: Analysis of variance
BM38, BM62 and *Blautia*: *Lachnospiraceae* strains
BMDC: Bone marrow-derived dendritic cell
*C. albicans*: *Candida albicans*
*C. trop*; *C. tropicalis*: *Candida tropicalis*
WTj: Wild-type mice purchased from Janvier Laboratory
WTj^WTj^: Wild-type mice from Janvier Laboratory transplanted with their own intestinal microbiota
WTj^D-1/2KO^: Wild-type mice from Janvier Laboratory transplanted with the intestinal microbiota of Dectin-1/2-deficient mice
CARD9: Caspase recruitment domain-containing protein 9
CFU: Colony-forming unit
D-1/2KO: Dectin-1 and Dectin-2KO mice
D-1/2KO^WTj^: Dectin-1/2-deficient mice transplanted with the intestinal microbiota of wild-type mice from Janvier Laboratory
D-1/2KO^D-1/2KO^: Dectin-1/2-deficient mice transplanted with their own intestinal microbiota
D-1KO: Dectin-1KO mice
D-2KO: Dectin-2KO mice
DAI: Disease activity index
DC: Dendritic cell
DSS: Dextran sodium sulfate
Fluco: Fluconazole
FMT: Fecal microbiota transfer
H&E: Hematoxylin and eosin
*H. capsulatum*: *Histoplasma capsulatum*
IBD: Inflammatory bowel disease
IFN: Interferon
IL-10: Interleukin 10
IL-6: Interleukin 6
KO: Knockout
LCN2: Lipocalin-2
LDA: Linear discriminant analysis
LEfSe: LDA effect size
LPS: Lipopolysaccharide
LYHBHI: Brain–heart infusion supplemented with cellobiose, maltose, and cysteine
*M. rest*; *M. restricta*: *Malassezia restricta*
mDixon: Modified Dixon
PBS: Phosphate-buffered saline
PCoA: Principal coordinates analysis
PEG: Polyethylene glycol
RPMI: Roswell Park Memorial Institute
S100a8: S100 Calcium Binding Protein A8
S100a9: S100 Calcium Binding Protein A9
SCFAs: Short-chain fatty acids
SEM: Standard error of the mean
SNP: Single-nucleotide polymorphism
Th17: T helper 17
TNFα: Tumor necrosis factor α
WT: Wild-type mice
YEPD: Yeast extract peptone dextrose
