# Supplementary figures and images for: Deletion of both Dectin-1 and Dectin-2 affects the bacterial but not fungal gut microbiota and susceptibility to colitis in mice
Source: Microbiome. 2022 Jun 14;10:91. doi: 10.1186/s40168-022-01273-4 (PMC9195441; doi:10.1186/s40168-022-01273-4)

Supp Figure 1

A

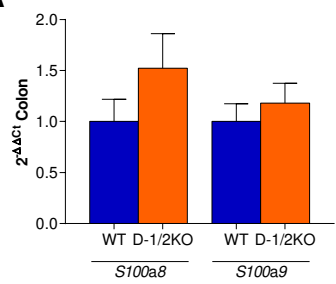

Supp Figure 2

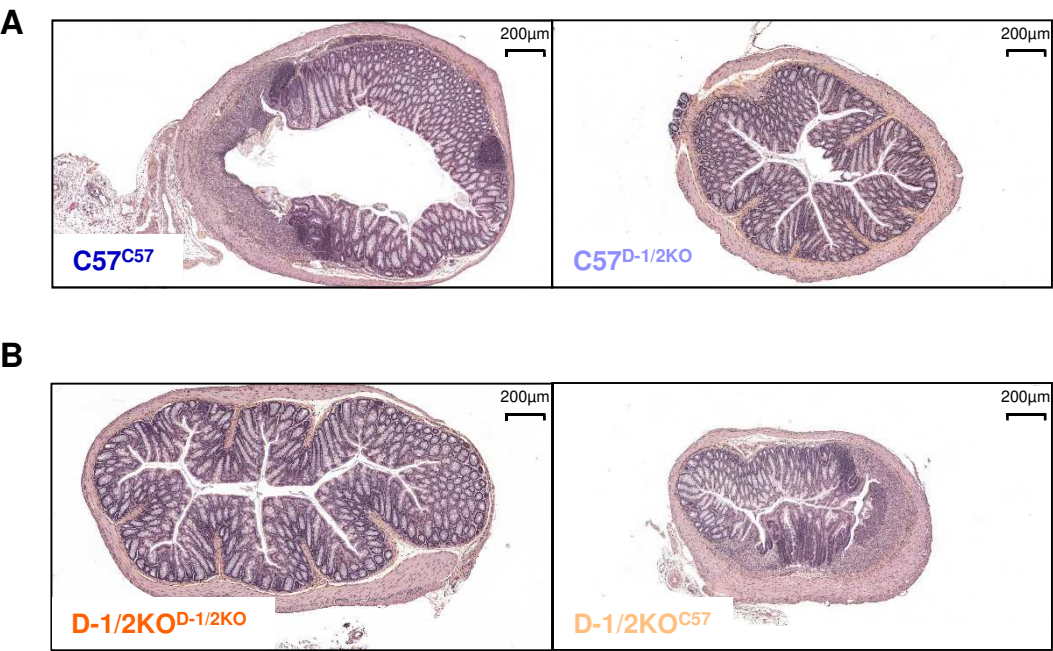

Supp Figure 3

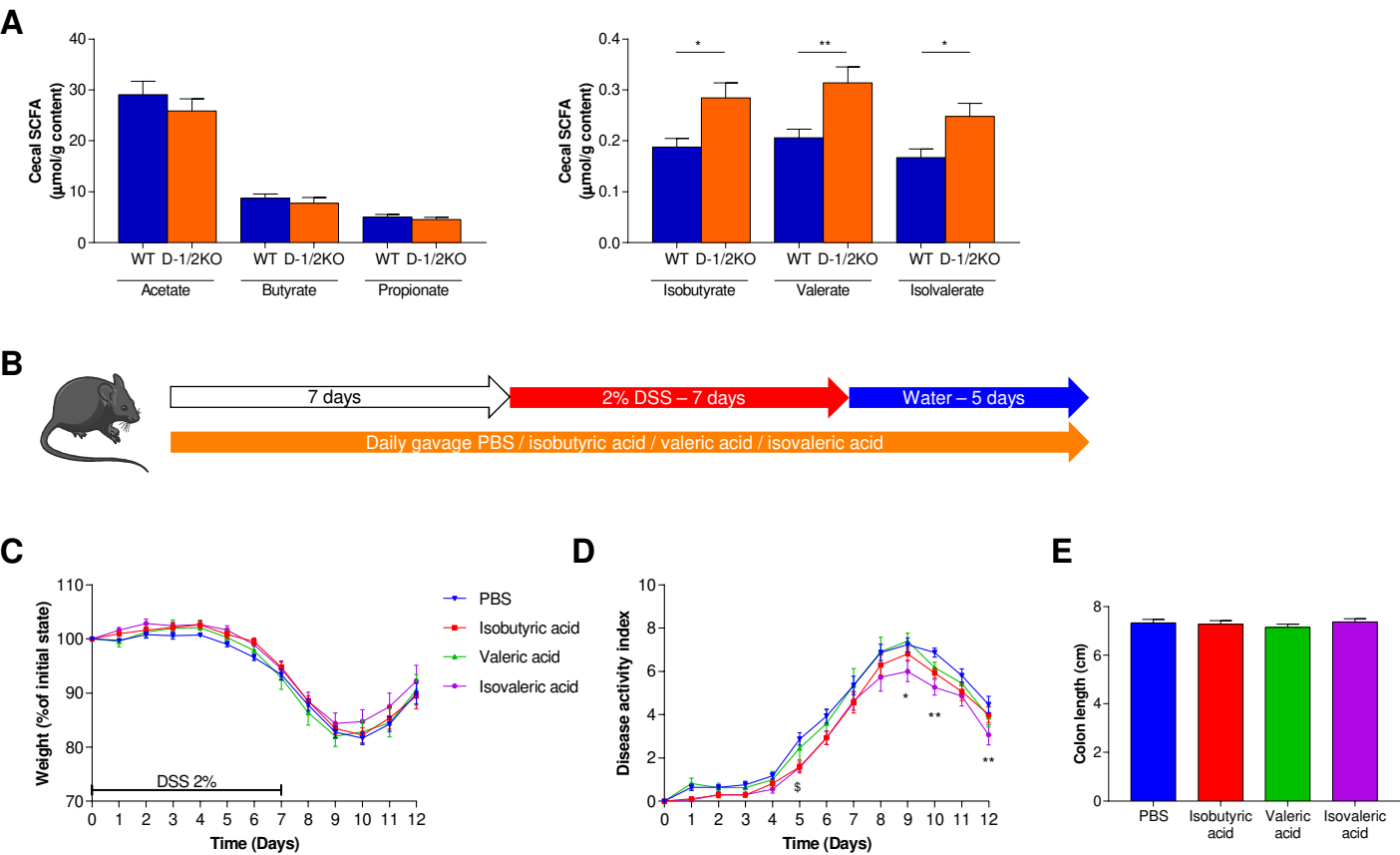

Supp Figure 4

A

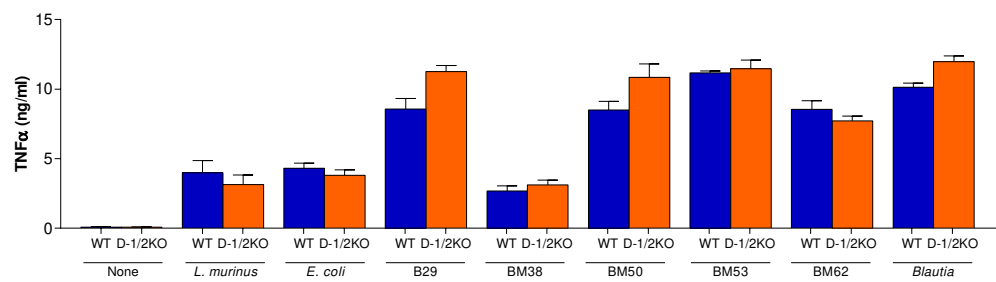

Supplement: Supplementary file 2 — Additional file 1: Supplemental Fig. 1. Antimicrobial peptides at baseline were not different. A. Antimicrobial peptides expressed in the colon of wild-type (WT) and Dectin-1/2-deficient (D-1/2KO) mice without challenge (qPCR). WT n = 8, D-1/2KO n= 9. Supplemental Fig. 2. Fecal microbiota transplantation either ameliorates or worsens DSS-induced colitis. (A) Wild-type mice from Janvier Laboratory transplanted with their own intestinal microbiota (WTjWTj) or transplanted with the intestinal microbiota of Dectin-1/2-deficient mice (WTjD-1/2KO) received dextran sulfate sodium (DSS) for 7 days. WTjWTj n = 18, WTjD-1/2KO n = 20. (B) Dectin-1/2-deficient mice transplanted with their own intestinal microbiota (D-1/2KOD-1/2KO) or transplanted with the intestinal microbiota of wild-type mice from Janvier Laboratory (D-1/2KOWTj) received dextran sulfate sodium (DSS) for 7 days. D-1/2KOD-1/2KO n = 17, D-1/2KOWTj n = 18. A-B. Representative H&E-stained images of proximal colon cross sections on Day 12 after initial DSS exposure. Supplemental Fig. 3. SCFA quantification and administration. A. Quantification of short-chain fatty acids (SCFAs) in the cecum of wild-type (WT) and Dectin-1/2-deficient (D-1/2KO) mice without challenge. WT n = 10, D-1/2KO n= 10. (B-E) Mice received several SCFAs (isobutyric acid, valeric acid or isovaleric acid) or vehicle (PBS) and dextran sulfate sodium (DSS) for 7 days. PBS n = 17, isobutyric acid n = 17, valeric acid n = 11, isovaleric acid n = 16. B. Experimental design for the administration of SCFAs and DSS. C. Weight of DSS-exposed mice. D. Disease activity index (DAI) of DSS-exposed mice. E. Length of the colons of mice treated with DSS. For statistical comparisons, (*) indicates isovaleric acid versus PBS, and ($) indicates isobutyric acid versus PBS. *,$p <0.05, **p <0.01. Supplemental Fig. 4. Lachnospiraceae strains co-culture with BMDC elicit different TNF-α production. A. ELISA of the expression of TNF-α in BMDC cultures from wild-type (W [file 40168_2022_1273_MOESM2_ESM.pdf]
