# Supplementary material for: Deletion of both Dectin-1 and Dectin-2 affects the bacterial but not fungal gut microbiota and susceptibility to colitis in mice
Source: Microbiome. 2022 Jun 14;10:91. doi: 10.1186/s40168-022-01273-4 (PMC9195441; doi:10.1186/s40168-022-01273-4)
Supplement: Supplementary file 3 — Additional file 2. [file 40168_2022_1273_MOESM3_ESM.docx]

**Supplemental Table 1**

| **Name** | **5’- Forward - 3’** | **5’- Reverse - 3'** |
| --- | --- | --- |
| All Bacteria | CGGTGAATACGTTCCCGG | TACGGCTACCTTGTTACGACTT |
| All-Bacteria Probe | 6FAM-CTTGTACACACCGCCCGTC-MGB | |
| All Fungi | ATTGGAGGGCAAGTCTGGTG | CCGATCCCTAGTCGGCATAG |
| Dectin-1 | GCCAATGCTGCCGACTCCAG | GCTGTAACTTCTGAAGAAAAC |
| Dectin-2 | AGGAATACGTACTGTGATGAGGATC | TAGAAATATTTCACCCTCAGGCAC |
| IL-10 | AGAAGCATGGCCCAGAAATCA | GGCCTTGTAGACACCTTGGT |
| IL-6 | GTAGCTATGGTACTCCAGAAGAC | ACGATGATGCACTTGCAGAA |
| *S100*a*8* | TCAAGACATCGTTTGAAAGGAAATC | GGTAGACATCAATGAGGTTGCTC |
| *S100*a*9* | AAAGGCTGTGGGAAGTAATTAAGAG | GCCATTGAGTAAGCCATTCCC |
